# Cost-Effectiveness of Polygenic Risk Score–Guided Statin Therapy for Cardiovascular Disease Prevention in Singapore

**DOI:** 10.1016/j.jacasi.2026.07.018

**Published:** 2026-08-27

**Authors:** Irene Li Peng Ooi, Aloysius Wei-Yan Chia, Ying Jie Chee, Liuh Ling Goh, Khai Pang Leong, Sanchalika Acharyya, Rinkoo Dalan, Akshar Saxena

**Affiliations:** a Division of Economics, Nanyang Technological University, Singapore, Singapore; b Department of Endocrinology, KLARIS Precision Health Research Clinic, Tan Tock Seng Hospital, NHG Health, Singapore, Singapore; c Molecular Diagnostic Laboratory, Tan Tock Seng Hospital, NHG Health, Singapore, Singapore; d Department of Rheumatology, Allergy and Immunology, Tan Tock Seng Hospital, NHG Health, Singapore, Singapore; e Lee Kong Chian School of Medicine, Nanyang Technological University, Singapore, Singapore

**Keywords:** cardiovascular disease prevention, cost-effectiveness, polygenic risk score, precision medicine, Singapore, statin therapy

## Abstract

**Background:**

Atherosclerotic cardiovascular disease (ASCVD) is a leading cause of morbidity and mortality in Singapore. Under current guidelines, individuals at intermediate risk often do not receive preventive statin therapy despite potentially elevated genetic risk. Polygenic risk scores (PRSs) offer an opportunity to refine risk stratification, but their economic value in Asian health care settings remains uncertain.

**Objectives:**

The authors aimed to evaluate the cost-effectiveness of PRS-guided risk assessment for a cohort of 45-year-old Singapore residents with intermediate (7.5%-20%) 10-year ASCVD risk.

**Methods:**

We developed a state-transition Markov model comparing 2 strategies: 1) standard risk assessment using the pooled cohort equation, with statin initiation based on traditional risk factors; and 2) PRS-guided reclassification, with statin therapy initiated for individuals in the top 20% of the PRS distribution. Model inputs were derived from the literature as well as local registry data. Outcomes were evaluated from the health care payer perspective over 2- to 40-year horizons. Deterministic and probabilistic sensitivity analyses were conducted.

**Results:**

PRS-guided therapy was not cost-effective over 2- to 20-year horizons. From 30 years onward, it became cost-effective (Singapore dollars [SGD]) at a willingness-to-pay threshold of SGD 75,000/quality-adjusted life year (QALY), with an incremental cost-effectiveness ratio of SGD 60,334/QALY at 30 years and SGD 23,667/QALY at 40 years. Probabilistic analysis showed a 53.5% probability of cost-effectiveness, with mean incremental cost and effectiveness of SGD 292 (95% uncertainty interval [UI]: –SGD 490 to SGD 1,052) and 0.010 QALYs (95% UI: –0.062 to 0.087), respectively.

**Conclusions:**

Incorporating PRS into ASCVD risk stratification for intermediate-risk individuals in Singapore may represent a cost-effective long-term prevention strategy as benefits of avoided ASCVD events accumulate.

Atherosclerotic cardiovascular disease (ASCVD) is a leading cause of morbidity and mortality in Singapore, accounting for nearly 1 in 3 deaths in 2024.^1^ Singapore's multiethnic population, comprising Chinese, Malay, and Indian communities, presents a unique context for cardiovascular risk management, as differences in genetic predispositions, lifestyle factors, and health behaviors across ethnic groups may influence both disease risk and the effectiveness of preventive strategies. This multiethnic complexity adds particular relevance to efforts aimed at refining individual-level risk stratification beyond conventional clinical tools.

Traditional risk stratification tools, such as the pooled cohort equations (PCEs), rely on clinical and lifestyle factors, including age, blood pressure, cholesterol levels, smoking status, and diabetes. Global guidelines on lipid management further recommend incorporating ASCVD “risk-enhancing factors,” such as family history of premature ASCVD, a history of preeclampsia, and elevated lipoprotein(a) concentration, when considering statin initiation for intermediate-risk patients.^2^, ^3^, ^4^, ^5^ However, neither PCE nor these risk-enhancing factors systematically capture inherited genetic susceptibility to ASCVD.^6^ As a result, individuals with high genetic risk who do not manifest conventional clinical risk factors may remain unidentified and may not be offered preventive statin therapy, a gap that may be especially consequential in multiethnic populations where genetic risk profiles differ across groups.^2^, ^3^, ^4^^,^^7^

Polygenic risk scores (PRSs), which aggregate the effects of multiple common genetic variants associated with disease risk, have emerged as a promising tool to refine cardiovascular risk stratification.^8^, ^9^, ^10^, ^11^ Studies have shown that individuals in the top quintile of the PRS distribution have twice the odds of developing ASCVD compared with those in lower quintiles,^12^ and they derive greater relative risk reduction from statin therapy.^13^ Importantly, recent evidence from large Asian cohorts supports the predictive value of PRS in non-European populations,^11^ and Singapore's National Precision Medicine Programme, including the ongoing SG100K initiative to build a 100,000-person Asian population reference cohort, reflects growing recognition of the need for population-specific genomic tools in preventive health.^14^^,^^15^

Despite this promise, the economic value of integrating PRS into preventive cardiovascular strategies remains uncertain, particularly in Asian health care settings. Genomic testing entails upfront costs, but it may enable earlier intervention and reduced downstream disease burden. These trade-offs are especially relevant in Singapore, where PRS-based risk stratification models are yet to be validated at scale and where health care resource allocation decisions require locally grounded evidence.

This study evaluates the cost-effectiveness of integrating PRS into ASCVD risk stratification in Singapore. Using a health economic modeling approach, we compare standard PCE-based assessment with a PRS-guided strategy among intermediate-risk individuals, a group most likely to benefit from genetic risk reclassification, given that current guidelines already recommend incorporating risk-enhancing factors when deciding on statin initiation in this group. We quantify the potential impact on health outcomes and health care costs from the health care payer perspective, with the aim of informing policy discussions around precision prevention and resource allocation in Singapore's health system.

## Methods

### Overview

Approval for this study was obtained from Tan Tock Seng Hospital Institutional Review Board. We developed a state-transition Markov model to project the health care costs and health outcomes associated with integrating the cardiovascular polygenic risk score (CVD-PRS) into the risk assessment among individuals at intermediate 10-year ASCVD risk. The target cohort consisted of 45-year-old Singapore residents, and the analysis adopted a health care payer perspective. The model compared 2 strategies: 1) standard care using the PCE alone, and 2) PRS-guided reclassification, with statin therapy initiated for individuals in the top 20% of the PRS distribution.^7^ Outcomes were evaluated over multiple time horizons ranging from 2 to 40 years. This study followed the CHEERS (Consolidated Health Economic Evaluation Reporting Standards) reporting guideline (Table 1).^16^

**Table 1:** CHEERS 2022 Checklist^16^

Topic	No.	Item	Location Where Item Is Reported
Title	1	Identify the study as an economic evaluation and specify the interventions being compared.	Title page
Abstract	2	Provide a structured summary that highlights context, key methods, results, and alternative analyses.	Abstract
Introduction			
Background and objectives	3	Give the context for the study, the study question, and its practical relevance for decision making in policy or practice.	Introduction
Methods			
Health economic analysis plan	4	Indicate whether a health economic analysis plan was developed and where available.	Not reported
Study population	5	Describe characteristics of the study population (such as age range, demographics, socioeconomic, or clinical characteristics).	Methods - Overview
Setting and location	6	Provide relevant contextual information that may influence findings.	Methods - Overview
Comparators	7	Describe the interventions or strategies being compared and why chosen.	Methods - Strategies
Perspective	8	State the perspective(s) adopted by the study and why chosen.	Methods - Analysis
Time horizon	9	State the time horizon for the study and why appropriate.	Methods - Analysis
Discount rate	10	Report the discount rate(s) and reason chosen.	Methods - Utility values and costs
Selection of outcomes	11	Describe what outcomes were used as the measure(s) of benefit(s) and harm(s).	Methods - Analysis
Measurement of outcomes	12	Describe how outcomes used to capture benefit(s) and harm(s) were measured.	Methods - Analysis
Valuation of outcomes	13	Describe the population and methods used to measure and value outcomes.	Methods - Analysis
Measurement and valuation of resources and costs	14	Describe how costs were valued.	Methods - Costs
Currency, price date, and conversion	15	Report the dates of the estimated resource quantities and unit costs, plus the currency and year of conversion.	Methods - Costs
Rationale and description of model	16	If modeling is used, describe in detail and why used. Report if the model is publicly available and where it can be accessed.	Methods - Model structure
Analytics and assumptions	17	Describe any methods for analyzing or statistically transforming data, any extrapolation methods, and approaches for validating any model used.	Methods, last paragraph
Characterizing heterogeneity	18	Describe any methods used for estimating how the results of the study vary for subgroups.	Not reported
Characterizing distributional effects	19	Describe how impacts are distributed across different individuals or adjustments made to reflect priority populations.	Methods - Analysis
Characterizing uncertainty	20	Describe methods to characterize any sources of uncertainty in the analysis.	Methods - Analysis
Approach to engagement with patients and others affected by the study	21	Describe any approaches to engage patients or service recipients, the general public, communities, or stakeholders (such as clinicians or payers) in the design of the study.	Not reported
Results			
Study parameters	22	Report all analytic inputs (such as values, ranges, references), including uncertainty or distributional assumptions.	Table 1
Summary of main results	23	Report the mean values for the main categories of costs and outcomes of interest and summarize them in the most appropriate overall measure.	Results
Effect of uncertainty	24	Describe how uncertainty about analytic judgments, inputs, or projections affect findings. Report the effect of choice of discount rate and time horizon, if applicable.	Results
Effect of engagement with patients and others affected by the study	25	Report on any difference patient/service recipient, general public, community, or stakeholder involvement made to the approach or findings of the study	Not reported
Discussion			
Study findings, limitations, generalizability, and current knowledge	26	Report key findings, limitations, ethical or equity considerations not captured, and how these could affect patients, policy, or practice.	Discussion
Other relevant information			
Source of funding	27	Describe how the study was funded and any role of the funder in the identification, design, conduct, and reporting of the analysis	Title page
Conflicts of interest	28	Report authors' conflicts of interest according to journal or International Committee of Medical Journal Editors requirements.	Title page

CHEERS = Consolidated Health Economic Evaluation Reporting Standards.

### Study population

The model simulated a hypothetical cohort of 45-year-old individuals in Singapore with an intermediate (7.5%-20%) 10-year risk of ASCVD based on the PCE.^2^ The cohort was assumed to be disease-free at baseline and representative of Singapore's resident population in terms of age and sex distribution.

### Strategies

Two strategies were compared: 1) Standard care: Risk assessment based solely on the PCE. Among individuals with intermediate 10-year ASCVD risk, we assumed that 52% would receive statin therapy under standard care, based on the proportion meeting statin-eligibility criteria through conventional risk-enhancing factors.^17^ 2) CVD-PRS–guided strategy: Individuals at intermediate risk per PCE were additionally stratified using the CVD-PRS. Individuals in the top 20% of the PRS distribution were reclassified as high risk and received statin therapy, with ongoing risk management in primary care. Individuals not classified as high risk continued to receive statins according to standard eligibility criteria based on conventional risk-enhancing factors.

### Model structure

We developed a state-transition (Markov) model in TreeAge Pro 2025 to estimate the long-term cost-effectiveness of incorporating the CVD-PRS into ASCVD risk stratification. We referenced similar studies from other countries that used the same approach.^18^, ^19^, ^20^ The target cohort comprised adults aged 45 years with a 10-year intermediate risk of ASCVD, defined using the PCE. We used a simulation model because real-world patient-level data were not available to examine the health and economic consequences of implementing the PRS in Singapore.

At model entry, the cohort was assumed to be free of ASCVD. In each annual cycle, individuals could experience a first myocardial infarction (MI) or ischemic stroke, a recurrent ASCVD event, or death, with death defined as the absorbing state (Figures 1 and 2). Both disease-specific and age-adjusted background mortality were incorporated. Health-state utilities and costs were assigned to each state to project quality-adjusted life years (QALYs) and costs across the model time horizons. Individuals classified as at high genetic risk and receiving statin therapy (CVD-PRS–guided strategy) were modeled with a reduced probability of ASCVD events.

**Figure 1:**
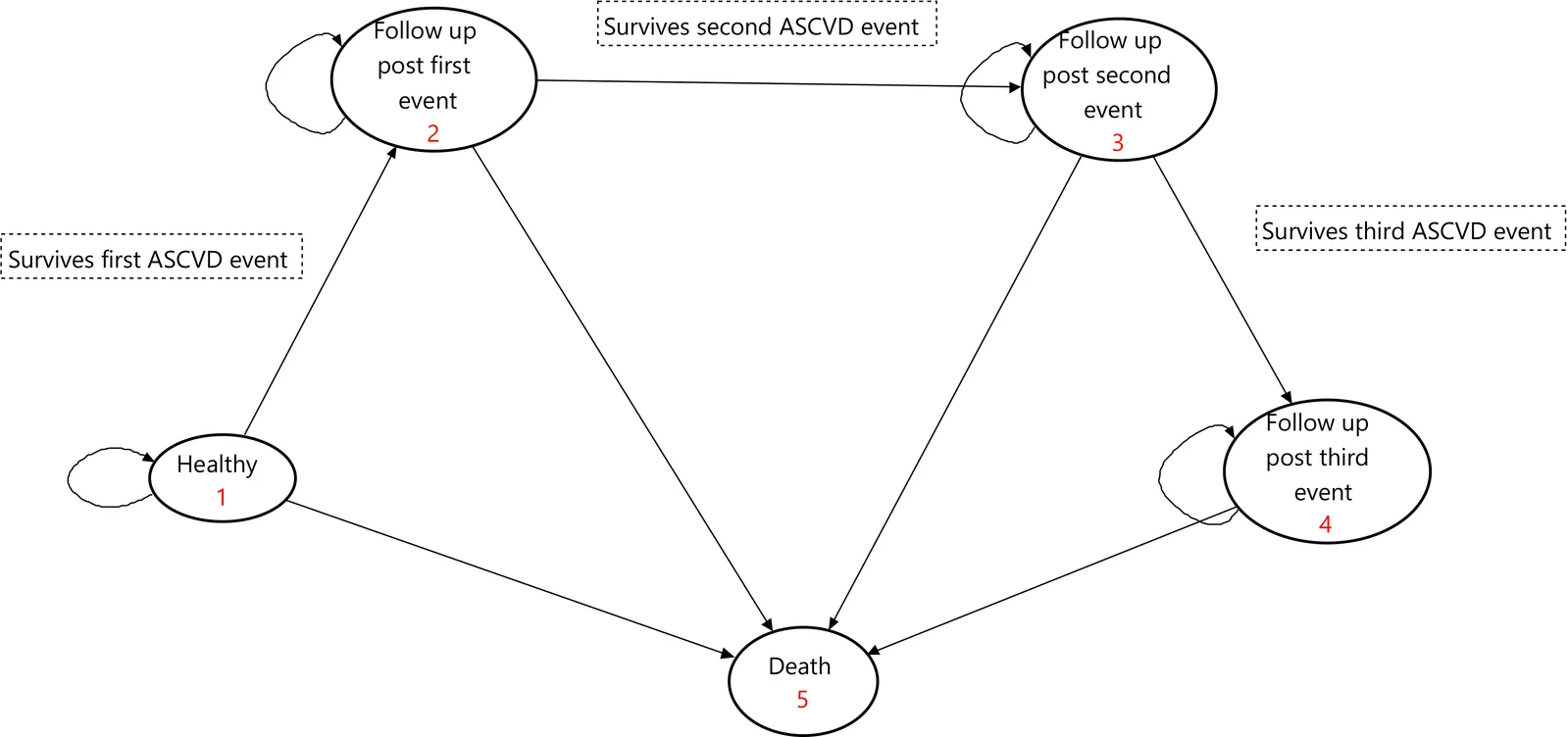
Health State Transition Diagram for the Markov Model This diagram illustrates the health states and permissible transitions in the Markov model. All individuals enter the model in the healthy (disease-free) state and may transition to the first ASCVD event (either myocardial infarction or stroke), followed by up to 2 recurrent ASCVD events, or to death. Death is the absorbing state and may occur from any health state. Both disease-specific and age-adjusted background mortality were incorporated. The cycle length was 1 year. Individuals receiving statin therapy under the PRS-guided strategy were modeled with a reduced probability of transitioning to an ASCVD event state. ASCVD = atherosclerotic cardiovascular disease; PRS = polygenic risk score.

**Figure 2:**
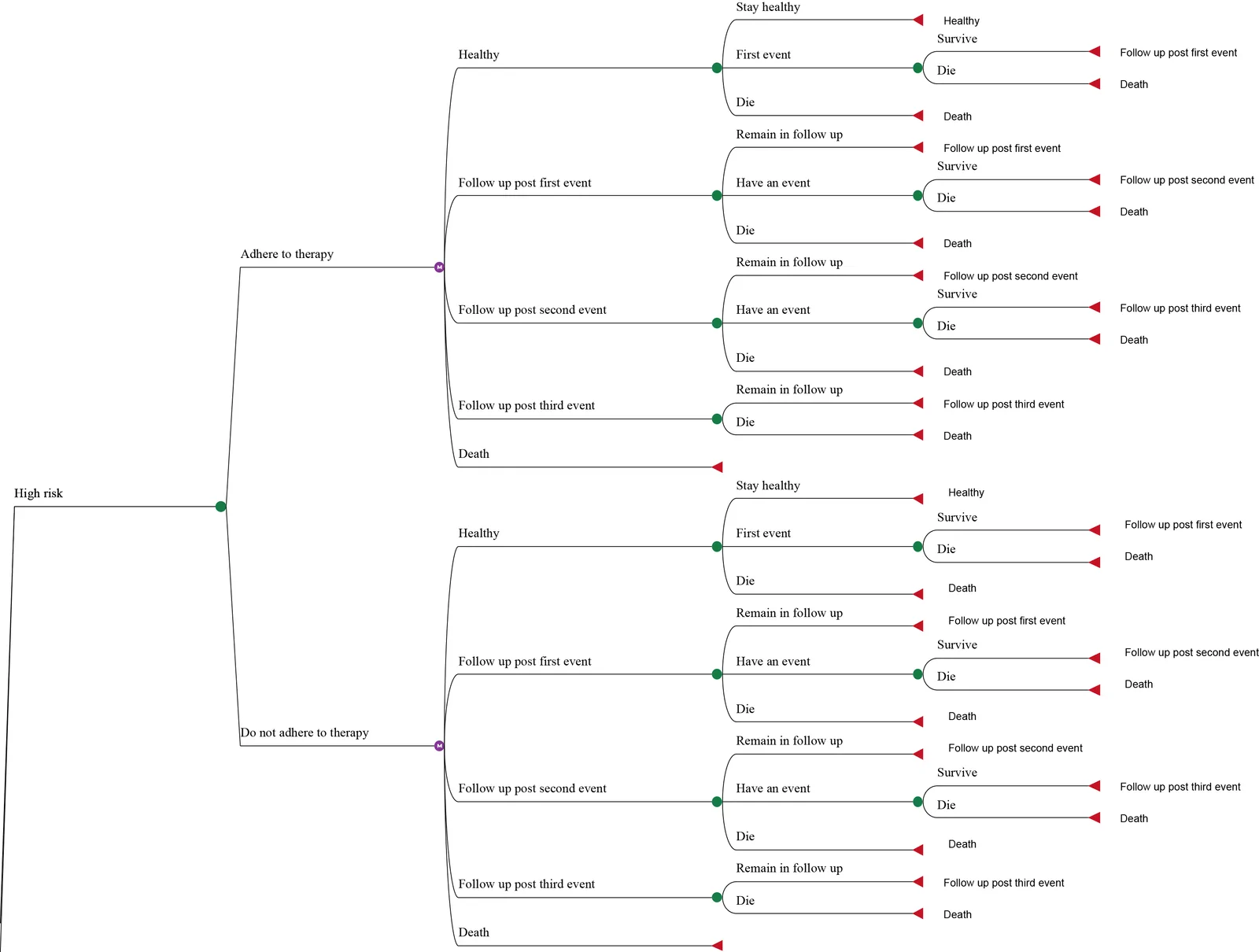
Markov-Tree Diagram for the PRS-Guided Strategy This diagram illustrates the decision pathway for an individual classified as being at high genetic risk under the PRS-guided strategy. At each annual cycle, the individual may experience a first ASCVD event, a recurrent ASCVD event, or remain event-free, with all-cause mortality incorporated throughout. In the base-case analysis, 57.5% of individuals eligible for statin therapy were assumed to remain adherent to treatment over the analytical time horizon. Statin use was modeled as reducing the probability of ASCVD events, which is reflected in the transition probabilities shown. Separate trees were constructed for individuals classified as non–high risk under the PRS-guided strategy. ASCVD = atherosclerotic cardiovascular disease; PRS = polygenic risk score.

### Parameter inputs

Model parameters were derived from published studies and are shown in Table 2. The baseline annual risk of developing ASCVD among individuals classified as intermediate risk by the PCE was obtained from the REGARDS (REasons for Geographic and Racial Differences in Stroke) study.^21^ REGARDS enrolled adults aged 45 to 79 years without ASCVD or diabetes, not taking statins, and with low-density lipoprotein cholesterol of 70 to 189 mg/dL. Outcomes of incident ASCVD included first MI or ischemic stroke events. For individuals in the top quintile of the CVD-PRS distribution, we applied a hazard ratio of 1.62 to reflect their higher genetic risk.^24^

**Table 2:** Parameter Inputs

	Base-Case Value	Source
Weightage to estimate mean: stroke	43.74%	National Registry of Diseases Office^52^^,^^53^
Weightage to estimate mean: MI	56.26%	National Registry of Diseases Office^52^^,^^53^
Transitional probabilities		
PRS high risk	0.2	Marston et al^7^
Adherence to treatment regime (high risk)	0.575	Basios et al^27^
Risk of ASCVD		
First ASCVD: intermediate PCE risk (no PRS)	0.010	Kong et al^54^
First ASCVD: intermediate PCE risk (no PRS), age 30-44 y group	0.00777	Calculated based on 10-y ASCVD risk of 7.5% (lower bound of intermediate-risk category)
First ASCVD: intermediate PREVENT risk (no PRS)	0.0164	Dhaliwal et al^43^
Hazard risk		
HR of ASCVD (high PRS risk)	1.62	Natarajan et al^24^
HR of ASCVD (high PRS risk)	1.84	Lu et al^44^
Post-ASCVD to another event	0.0397	An et al^55^
Statin effectiveness		
Intermediate-risk patients eligible for statins	0.5204	Hammond et al^17^
Relative risk of developing ASCVD on statin (intermediate-risk individuals)	0.69	Shepherd et al^56^
Relative risk of developing ASCVD on statin (high PRS)	0.56	Natarajan et al^24^
Relative risk of developing ASCVD on statin (non–high PRS)	0.76	Natarajan et al^24^
Mortality		
Acute MI to death	0.083	National Registry of Diseases Office^53^
Acute stroke to death	0.075	National Registry of Diseases Office^52^
Post-MI to death	0.02814	Velagaleti et al^57^
Poststroke to death	0.0413	Saposnik et al^58^
Risk of death, event-free	Age dependent	Registry of Births and Deaths Immigration & Checkpoints Authority Singapore^1^
Utility		
Stroke	0.64	Luengo-Fernandez et al^28^
Poststroke	0.66	Luengo-Fernandez et al^28^
MI	0.704	Sullivan and Ghushchyan^29^
Post-MI	0.88	Tsevat et al^30^
Disease free, on statins	0.99	Hutchins et al^59^
Costs (SGD)		
Cost of PRS testing	100	Information from Tan Tock Seng Hospital
Cost of statins	146	Information from Tan Tock Seng Hospital
Cost of consultation at polyclinics	102	NHG Polyclinics^60^
Stroke		
Nonfatal stroke (first year)	22,842	Ni et al,^32^ Goyal et al^33^
Poststroke (subsequent years)	10,724	Ni et al,^32^ Goyal et al^33^
Fatal stroke	6,666	Ni et al^32^
Myocardial infarction		
Nonfatal MI (first year)	33,641	Chin et al^34^
Post-MI (subsequent years)	949	Chin et al^34^
Fatal MI	9,883	Zhao et al^61^
Weighted cost of nonfatal ASCVD (first year)	28,918	Calculated
Weighted cost of post-ASCVD (subsequent year)	5,224	Calculated
Weighted cost of fatal ASCVD	8,476	Calculated

ASCVD = atherosclerotic cardiovascular disease; MI = myocardial infarction; PCE = pooled cohort equation; PREVENT = Predicting Risk of cardiovascular disease EVENTs; PRS = polygenic risk score; SGD = Singapore dollars.

Statins are recommended for the primary prevention of ASCVD events in high-risk individuals, and they have been shown to reduce ASCVD risk by 44% (HR: 0.56, 95% CI: 0.40-0.78; *P* < 0.001) among those at high genetic risk.^24^ As the efficacy of statins among moderate-risk individuals with high CVD-PRS has not been examined, we used the efficacy of pravastatin 40 mg daily from a study in high-risk, high CVD-PRS populations.^24^ Atorvastatin is one of the commonly prescribed statins in Singapore, and it has been shown to have comparable effectiveness in reducing low-density lipoprotein cholesterol.^25^^,^^26^ For modeling purposes, we applied separate statin treatment effects for high-PRS and non–high-PRS individuals, based on published evidence demonstrating differential treatment benefit across genetic-risk strata. In the base-case analysis, we assumed that 57.5% of the individuals identified as eligible for preventive therapy would remain adherent to statin therapy over the time horizon of the analysis.^27^ Lastly, we assumed that an individual could have up to 3 ASCVD events over the analytical time horizon.

### Utility values

Utility weights were assigned to reflect health-related quality of life associated with each health state in the model, with 1.0 representing perfect health and 0.0 representing death. Utility weights for MI, stroke, post-MI, and poststroke were derived from the literature. A weighted average of the utility values for MI and stroke was used to represent overall utility during ASCVD events and the post-ASCVD period.^28^, ^29^, ^30^ For individuals in the disease-free state receiving statin therapy, we incorporated a utility decrement to reflect the disutility of daily medication use. Based on evidence from the literature, the mean utility of taking a daily pill ranges from 0.990 to 0.994, and we conservatively applied the lowest estimate (0.990) in the base case.^31^ All utilities were discounted by 3% each year, in line with recommended economic evaluation guideline and consistent with guidelines used by the Agency for Care Effectiveness for Singapore's health technology assessments.

### Costs

Direct medical costs included PRS testing, statin therapy, routine outpatient consultations, and the acute and long-term management of ASCVD events. Cost estimates for ASCVD events were derived from published Singapore-specific studies and administrative data, with distinct methodologies applied for stroke and MI. These estimates included costs for hospitalization, procedures, medications, and postevent care. All costs were reported in 2024 Singapore dollars (SGD) and were discounted at an annual rate of 3%, in accordance with standard economic evaluation guidelines.

The unit cost of PRS testing was SGD 100, while the annual cost of statin therapy (atorvastatin) was SGD 146. Individuals receiving statins were assumed to attend a primary care physician consultation every 9 months, costing SGD 102 per visit based on polyclinic (public sector primary care) rates.

For stroke, total health care costs per patient included both the 90-day short-term health care cost after initial ischemic stroke and lifetime follow-up costs.^32^^,^^33^ The resulting estimates were SGD 22,842 in the first year and SGD 10,724 annually thereafter. Fatal stroke was assigned a 1-time cost of SGD 6,666.

For nonfatal MI, cost estimates were adapted from a previously published cost-effectiveness analysis of ticagrelor in patients with acute coronary syndromes, in which a 2-step approach was used to address the limited availability of comprehensive cost data in Singapore.^34^ First, acute hospitalization costs were obtained from the public hospital accounting system using diagnosis-related group codes corresponding to MI. These costs were averaged across hospitals and were weighted by the number of patients within each diagnosis-related group. Second, a South Korean report was used to estimate the relative proportions of hospital versus outpatient costs in the first and subsequent years following MI.^35^ These relative cost ratios were then applied to the Singapore acute hospitalization estimates to impute total health care costs, including both inpatient and outpatient components, for the corresponding time periods. These estimates thus included costs for drugs, revascularization procedures, hospitalization, physician visits, and echocardiography, radiography, and laboratory investigations. The resultant costs were SGD 33,641 for the first year after the MI event and SGD 949 annually for subsequent years. The cost for fatal MI was assumed to be the same as the cost of treating an episode of MI and was assigned a 1-time cost of SGD 9,883.^36^

Lastly, we calculated the weighted average costs for ASCVD events based on the incidence distribution of MI (56.3%) and stroke (43.7%) in Singapore.^37^^,^^38^ Age-specific mortality rates were derived from the Department of Statistics Singapore.^39^

### Analysis

We conducted the cost-effectiveness analysis from the Singapore public health care payer perspective, comparing total costs and health outcomes for the PRS-guided strategy versus standard PCE-based assessment across multiple time horizons: 2 years, 5 years, 10 years, 20 years, 30 years, and 40 years. Outcomes included total and incremental costs, total and incremental QALYs, incremental cost-effectiveness ratios (ICERs), and net monetary benefit (NMB).

The World Health Organization recommends using thresholds of 1 to 3 times the country's annual gross domestic product per capita for assessing cost-effectiveness.^40^ A strategy was considered cost-effective if the ICER was below 1 times the gross domestic product per capita threshold (SGD 120,000 for Singapore in 2024). Given that this may overstate affordability, a more conservative willingness-to-pay (WTP) threshold of SGD 75,000/QALY was applied, consistent with thresholds commonly used by the Agency for Care Effectiveness, Singapore's health technology assessment body.^41^ A review of the Agency for Care Effectiveness's drug and device appraisals found that most drugs with ICERs below SGD 75,000/QALY received positive recommendations from the agency.^42^

We assessed external consistency by comparing projected outcomes under the standard care as well as the magnitude and direction of the incremental costs and effectiveness with published data from other modeling and clinical studies.^10^^,^^18^

### Scenario analysis and sensitivity analysis

Scenario analyses examined the impact of several variables, as listed below.

1) Changing the ASCVD event rate due to statin eligibility with the PREVENT (Predicting Risk of cardiovascular disease EVENTs) equation instead of PCE.^43^ We repeated the analysis using the PREVENT equation in place of PCE to examine how the choice of risk equation affected cost-effectiveness results.
2) Lowering the starting age of the cohort to 30 years: We assumed that ASCVD incidence between ages 30 to 44 would be lower than in the base-case cohort aged ≥45 years. Because age-specific ASCVD incidence estimates for an intermediate-risk Singapore cohort were not available, we approximated risk in this subgroup using the lower bound of the intermediate 10-year risk category (7.5%) to derive the corresponding annual event probability. This assumption was intended to reflect the lower expected event burden at younger ages in Asian populations^22^^,^^23^ while retaining consistency with the modeled intermediate-risk population.
3) Substituting the HR of having an ASCVD event for high–PRS risk individuals with the estimate reported in a large Chinese prospective cohort.^44^

We conducted 1-way deterministic sensitivity analysis to examine how variation in individual model parameters influenced the incremental NMB. We varied a single parameter at a time while keeping all other parameters at their baseline values. The plausible ranges for each parameter were derived from published studies where available. Because the MI cost estimates were partly derived using a nonlocal cost ratio, we used a wider variance (±50%) to reflect uncertainty associated with the cross-country transfer of cost inputs. Results were summarized using a tornado diagram to show the relative influence of each parameter on the incremental NMB.

We also performed probabilistic sensitivity analysis (PSA) to quantify the combined impact of uncertainty in model parameters on the incremental NMB. We assigned beta distributions to probability parameters, log-normal distributions to relative risk and hazard ratios, and gamma distributions to cost parameters, as shown in Table 3. We performed 10,000 Monte Carlo simulations and plotted the resulting incremental cost-effectiveness pairs on the cost-effectiveness plane. We also generated a cost-effectiveness acceptability curve to show the probability that the PRS-guided strategy would be cost-effective across a range of WTP thresholds.

**Table 3:** Model Parameters With Ranges Used for Sensitivity Analyses

	Base-Case Value	Lower Bound	Upper Bound	Distribution
Transitional probabilities				
PRS high risk	0.2	0.15	0.25	Beta
Adherence to treatment regime	0.575	0.486	0.663	Beta
Risk of ASCVD				
First ASCVD - Intermediate PCE risk	0.010	0.005	0.020	Beta
HR of ASCVD for top quintile of PRS	1.62	1.29	2.05	Log normal
Post-ASCVD to another event	0.040	0.0286	0.0518	Beta
Statin effectiveness				
Intermediate risk patients who are eligible for statins	0.5204	0.4163	0.6245	Beta
RR of developing ASCVD on statin (intermediate risk)	0.69	0.57	0.83	Log normal
RR of developing ASCVD on statin (high PRS)	0.56	0.40	0.78	Log normal
RR of developing ASCVD on statin (non–high PRS)	0.76	0.63	0.92	Log normal
Mortality				
Acute MI	0.08	0.066	0.100	Beta
Acute stroke	0.08	0.059	0.091	Beta
Post-MI	0.04	0.026	0.049	Beta
Poststroke	0.04	0.029	0.053	Beta
Utility				
Stroke	0.64	0.512	0.768	Beta
Poststroke	0.66	0.528	0.792	Beta
MI	0.70	0.600	0.810	Beta
Post-MI	0.88	0.840	0.930	Beta
Disease free, on statins	0.99	0.987	0.992	Beta
Costs (SGD)				
PRS testing	100	75	125	Gamma
Statins	146	110	183	Gamma
Consultation	102	77	128	Gamma
Stroke				
Nonfatal stroke (first year)	22,842	17,132	28,553	Gamma
Poststroke (subsequent years)	10,724	8,043	13,404	Gamma
Fatal stroke	6,666	4,999	8,332	Gamma
Myocardial infarction				
Nonfatal MI (first year)	33,641	16,821	50,462	Gamma
Post-MI (subsequent years)	949	475	1,424	Gamma
Fatal MI	9,883	7,412	12,354	Gamma

Abbreviations as in Table 2.

To address potential correlation among PSA inputs, we reviewed model parameters for structural dependence and shared sources of uncertainty.^45^ Because empirical covariance matrices were not available for the key relative risk and cost inputs, we did not impose a full multivariate correlation structure, as specifying arbitrary pairwise correlations could introduce unsupported assumptions. Instead, we implemented a correlated-uncertainty scenario using common multiplicative scaling factors for parameter groups expected to move together. Specifically, a log-normal multiplier (μ = 0, σ = 0.084) was applied jointly to statin treatment-effect parameters, corresponding to a median multiplier of 1.00 and approximately −15% to +18% variation around the median. Separate log-normal multipliers with the same parameterization were applied to MI and stroke cost families, so that acute, fatal, and follow-up costs within each disease category varied together within each PSA iteration. We then reran the PSA and compared mean incremental costs, incremental QALYs, 95% uncertainty intervals (95% UIs), and the cost-effectiveness acceptability results with those from the primary PSA.

## Results

Table 4 presents our main findings and shows that in the short term (2- to 20-year time horizons), the standard care (no PRS) strategy dominated, reflecting higher upfront costs associated with PRS testing and statin initiation. At these time horizons, the PRS strategy generated slightly lower QALYs and higher total costs, and therefore was not cost-effective relative to standard care.

**Table 4:** Cost-Effectiveness Rankings for Base-Case Analysis

Time Horizon	Strategy	Cost (SGD)	Incremental Cost (SGD)	QALYs	Incremental QALYs	ICER	Net Monetary Benefit (SGD)
2 years	No PRS (standard care)	720	—	1.924	—	—	143,541
	PRS	842	123	1.923	−0.001	Dominated^a^	143,342
5 years	No PRS (standard care)	2,024	—	4.575	—	—	341,080
	PRS	2,175	151	4.572	−0.002	Dominated	340,761
10 years	No PRS (standard care)	4,532	—	8.415	—	—	626,619
	PRS	4,717	185	8.412	−0.003	Dominated	626,180
20 years	No PRS (standard care)	9,636	—	14.215	—	—	1,056,467
	PRS	9,862	227	14.213	−0.002	Dominated	1,056,107
30 years	No PRS (standard care)	13,808	—	17.960	—	—	1,333,224
	PRS	14,062	254	17.964	0.004	60,334	1,333,286
40 years	No PRS (standard care)	16,650	—	20.051	—	—	1,487,179
	PRS	16,925	276	20.063	0.012	23,667	1,487,777

ICER = incremental cost-effectiveness ratio; PRS = polygenic risk score; QALY = quality-adjusted life year; SGD = Singapore dollars.

a PRS was considered dominated where it was both more costly and generated fewer QALYs than standard care; the QALY differences at these horizons are small in magnitude and reflect modeling uncertainty.

From the 30-year time horizon onward, however, the PRS-guided strategy became cost-effective compared to standard care. At 30 years, the ICER was SGD 60,334/QALY, declining to SGD 23,667/QALY at 40 years as the cumulative benefits of avoided ASCVD events accrued over time.

When the PREVENT equation was used for risk stratification instead of PCE, the PRS strategy yielded an ICER of SGD 6,204/QALY compared to standard care, reflecting both lower incremental costs and greater incremental QALY gains than in the base-case analysis (Tables 5 and 6). In the scenario analysis with PRS testing initiated at age 30, the ICER was SGD 15,893/QALY over a 55-year time horizon, such that the cohort reached the same end age as in the base-case analysis. In the third scenario analysis substituting the HR for individuals with high-PRS ASCVD risk, the PRS strategy yielded a higher incremental cost and lower incremental effectiveness compared to the base-case scenario, with an ICER of SGD 49,724/QALY.

**Table 5:** Cost-Effectiveness Rankings for Scenario Analysis Compared to Base Case

Scenario	Incremental Cost	Incremental Effectiveness	ICER
1) Base-case analysis	SGD 276	0.012	SGD 23,667/QALY
2) PREVENT equation used to estimate risk of ASCVD and statin eligibility instead of PCE	SGD 175	0.028	SGD 6,204/QALY
3) Starting age of cohort lowered to 30 years, and time horizon extended to 55 years	SGD 330	0.021	SGD 15,893/QALY
4) Substituting the HR of having an ASCVD event for high-PRS risk individuals with the estimate reported in a large Chinese prospective cohort (HR: 1.84)	SGD 330	0.007	SGD 49,724/QALY
5) Correlated-uncertainty scenario using common multiplicative scaling factors	SGD 292	0.0098	SGD 29,745/QALY

ASCVD = atherosclerotic cardiovascular disease; ICER = incremental cost-effectiveness ratio; PCE = pooled cohort equation; PREVENT = Predicting Risk of cardiovascular disease EVENTs; PRS = polygenic risk score; QALY = quality-adjusted life year; SGD = Singapore dollars.

**Table 6:** Comparison of Results Between Base-Case Analysis and Scenario Analysis With PREVENT Equation Instead of PCE

Metric	PCE	PREVENT
Baseline ASCVD event risk	0.010	0.0164
Events prevented	403 per 100,000	712 per 100,000
ASCVD deaths prevented	32 per 100,000	56 per 100,000
Incremental cost	SGD 276	SGD 175
Incremental QALYs	0.012	0.028
ICER	SGD 23,667/QALY	SGD 6,204/QALY

ASCVD = atherosclerotic cardiovascular disease; ICER = incremental cost-effectiveness ratio; PCE = pooled cohort equation; PREVENT = Predicting Risk of cardiovascular disease EVENTs; QALY = quality-adjusted life year; SGD = Singapore dollars.

Figure 3 shows the results of the 1-way sensitivity analysis of variations in the transition probabilities, effect of statins, adherence to statin therapy, utility values, and costs. The tornado diagram showed that the parameters exerting the greatest influence on incremental NMB were the relative risk of ASCVD under statin therapy among intermediate-risk individuals, the corresponding relative risks for PRS high-risk and PRS non–high-risk individuals, the baseline probability of ASCVD events in the intermediate-risk cohort, and adherence to statin therapy. The NMB decreased below SGD 0 when the relative risk of intermediate-risk patients on statin therapy decreased below 0.674, indicating that the PRS-guided strategy was no longer cost-effective. Similarly, the PRS strategy was dominated by standard care when the relative risks for PRS high-risk and PRS non–high-risk individuals exceeded 0.588 and 0.782, respectively. When the incidence rate of the ASCVD event for individuals with intermediate risk fell below 0.007, the PRS strategy was dominated by standard care.

**Figure 3:**
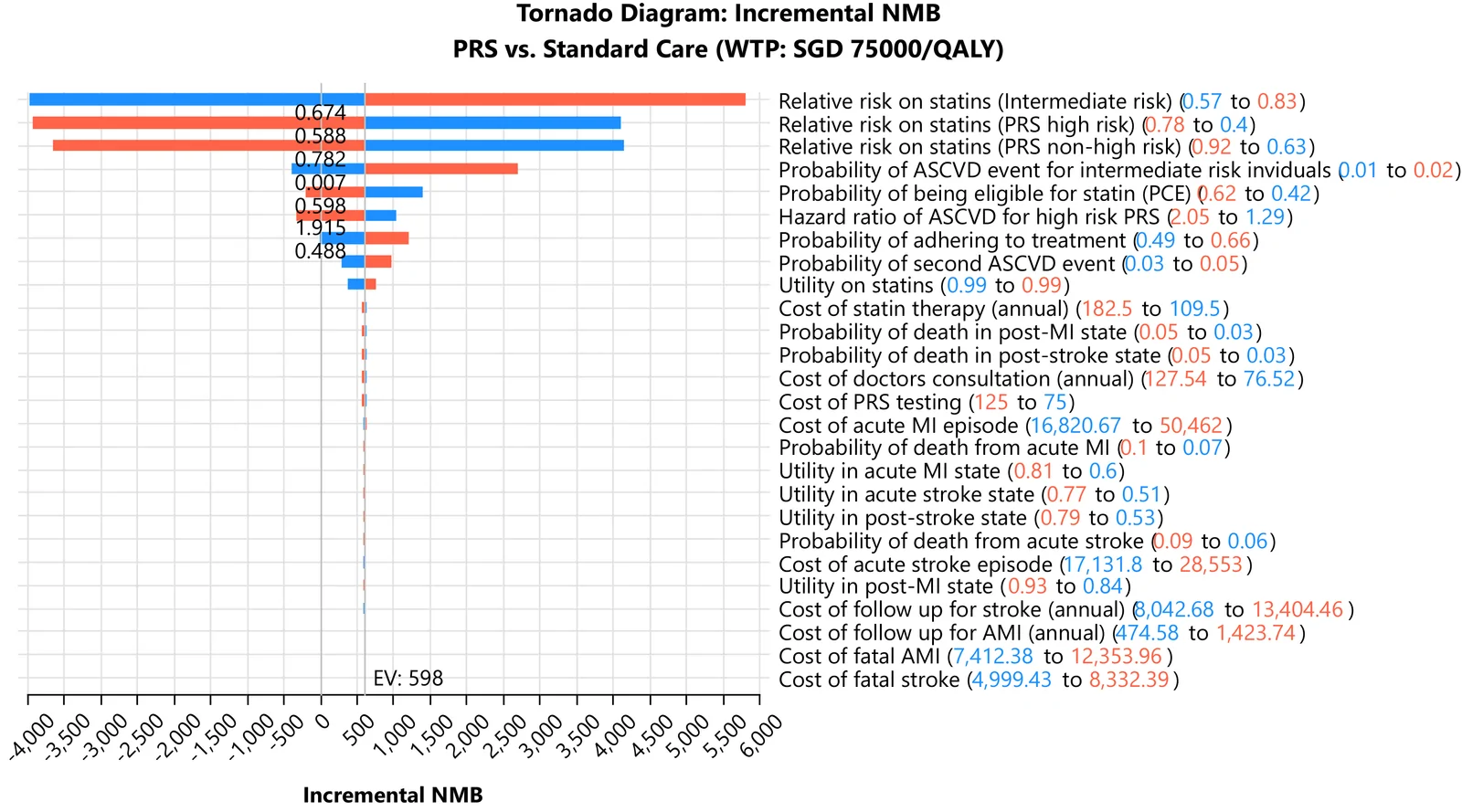
One-Way Sensitivity Analysis of Key Model Parameters This tornado diagram presents the results of 1-way deterministic sensitivity analyses examining the influence of individual model parameters on the incremental net monetary benefit (NMB) of the PRS-guided strategy versus standard care at the 40-year time horizon. The length of each bar indicates the range of NMB values when the corresponding parameter was varied between its lower and upper bounds, with the midline representing the base-case value. Parameters are ranked according to the magnitude of their influence. PRS = polygenic risk score; QALY = quality-adjusted life year; SGD = Singapore dollars; WTP = willingness to pay.

Figures 4 and 5 show that when using 10,000 Monte Carlo simulations and a WTP threshold of SGD 75,000 per QALY, the PRS strategy had a 53.5% probability of being cost-effective compared with standard care. The mean incremental cost and effectiveness from the probabilistic sensitivity analysis were SGD 292 (95% UI: SGD 490 to SGD 1,052) and 0.010 QALYs (95% UI: −0.062 to 0.087), respectively. The cost-effectiveness acceptability curve (Figure 5) further demonstrates that this probability exceeded 50% at a WTP threshold of approximately SGD 37,500/QALY, well below the reference threshold of SGD 75,000/QALY.

**Figure 4:**
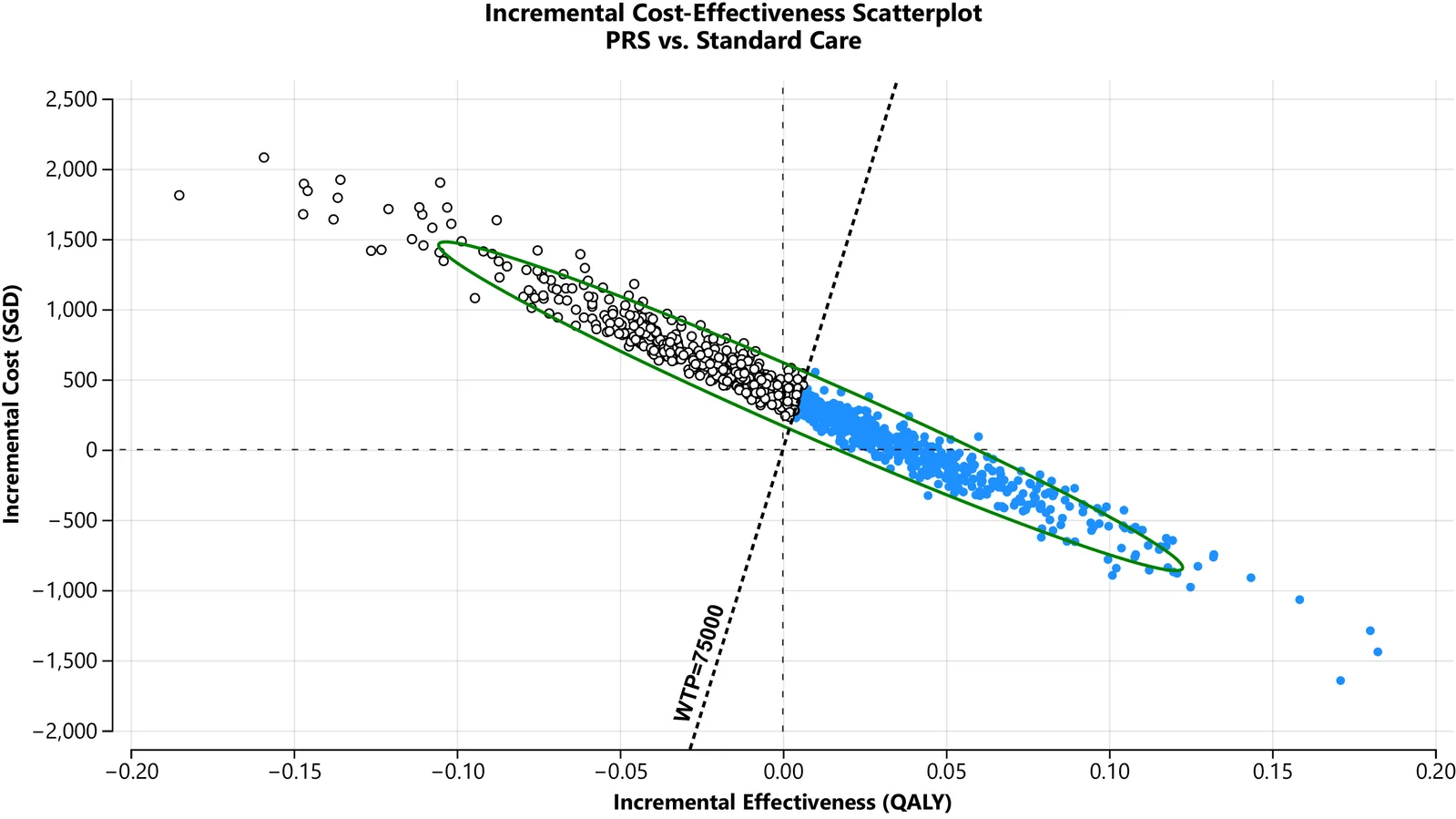
Cost-Effectiveness Plane From Probabilistic Sensitivity Analysis This scatter plot presents the results of the probabilistic sensitivity analysis based on 10,000 Monte Carlo simulations. Each point represents 1 simulation, plotting the incremental cost against the incremental quality-adjusted life years of the PRS-guided strategy relative to standard care. The blue triangles represent the PRS strategy, and the white circles represent standard care without PRS testing. The dotted line represents the nominal acceptable willingness-to-pay of SGD 75,000 per QALY. Points below this line represent simulations in which the PRS-guided strategy was cost-effective at that threshold. The distribution of points illustrates the uncertainty surrounding the incremental costs and QALYs associated with PRS-guided statin initiation. PRS = polygenic risk score; QALY = quality-adjusted life year; SGD = Singapore dollars; WTP = willingness-to-pay.

**Figure 5:**
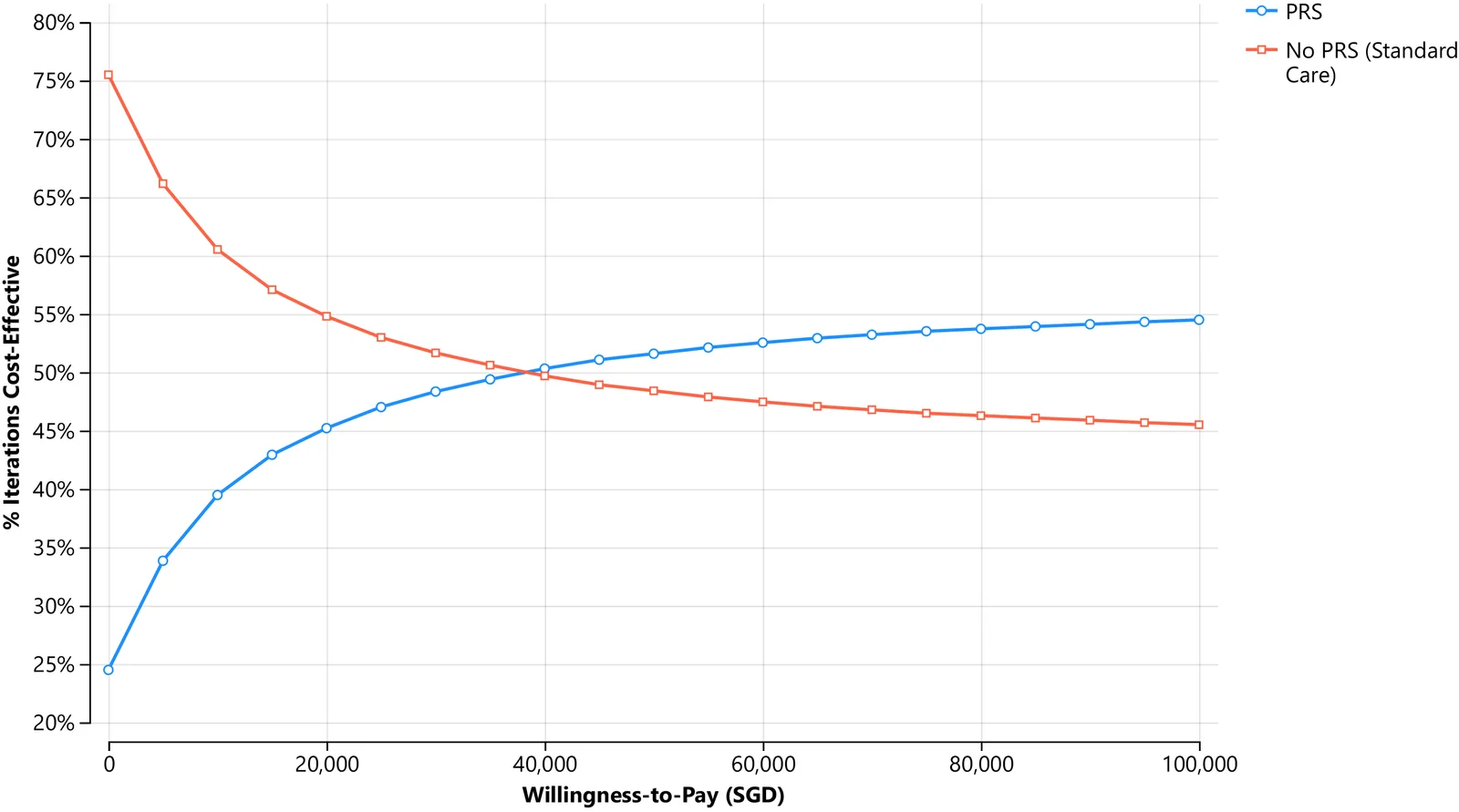
Cost-Effectiveness Acceptability Curve Based on 10,000 iterations, the curve represents the estimated probability that the PRS strategy would be deemed cost-effective at different willingness-to-pay (WTP) threshold levels. At a WTP of SGD 75,000, the PRS strategy has a 53.5% probability of being cost-effective. PRS = polygenic risk score; SGD = Singapore dollars.

In the correlated-uncertainty PSA scenario, PRS was associated with a mean incremental cost of SGD 292 (95% UI: −SGD 698 to SGD 1,238) and a mean incremental QALY gain of 0.00980 (95% UI: −0.08062 to 0.10742), yielding an ICER of SGD 29,745 per QALY gained. At a WTP threshold of SGD 75,000/QALY, the probability that PRS was cost-effective was 53.7%. These results were consistent with the primary PSA findings, with the ICER remaining well below the SGD 75,000/QALY threshold and the probability of cost-effectiveness changing by 0.2 percentage points.

## Discussion

### Principal findings

We developed a Markov model to evaluate the cost-effectiveness of integrating PRS with PCE for guiding statin initiation among adults at intermediate ASCVD risk in Singapore (Central Illustration). Our results suggest that PRS-guided therapy was not cost-effective in the short term (2-20 years), reflecting the upfront costs of PRS testing and preventive treatment. From the 30-year horizon onward, however, the PRS-guided strategy became cost-effective compared with standard care at a WTP threshold of SGD 75,000/QALY. Over the 40-year horizon, PRS-guided statin initiation resulted in an incremental cost of SGD 276 and a gain of 0.012 QALYs per person compared with standard PCE-based assessment, with an ICER of SGD 23,667/QALY.

**Central Illustration:**
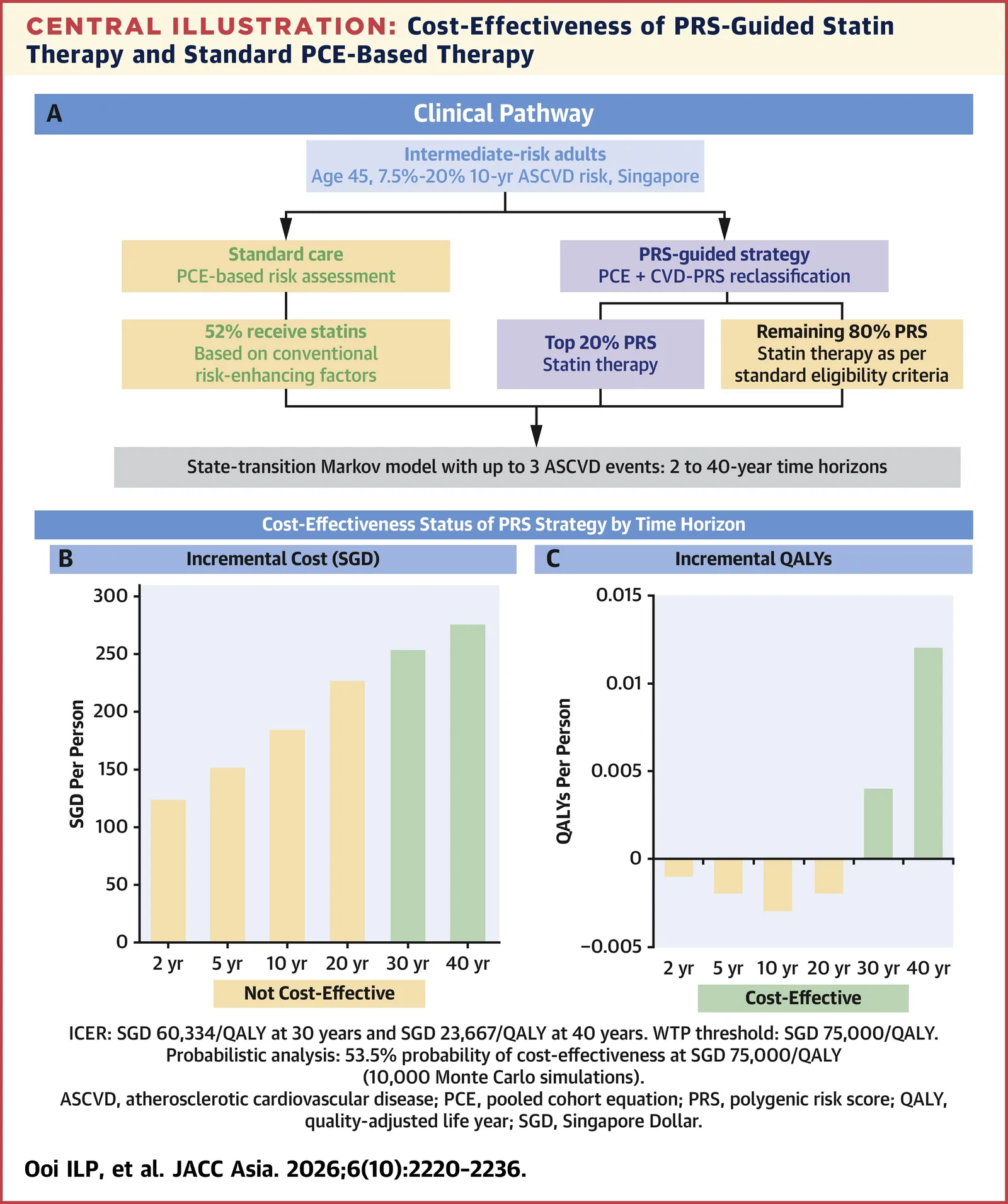
Cost-Effectiveness of PRS-Guided Statin Therapy and Standard PCE-Based Therapy (A) The clinical decision pathway is illustrated for intermediate-risk adults (7.5%-20% 10-year ASCVD risk at age 45) comparing standard PCE-based care, in which 52% of individuals initiate statins based on risk-enhancing factors, against a PRS-guided strategy that reclassifies individuals in the top 20% of the polygenic risk score distribution as high-risk and initiates statin therapy accordingly. Both strategies were modeled in a state-transition Markov model allowing up to 3 ASCVD events over time horizons ranging from 2 to 40 years. (B and C) The incremental costs (in Singapore dollars [SGD]) and incremental QALYs of the PRS-guided strategy versus standard care by time horizon. The amber bars indicate time horizons at which PRS was not cost-effective, whereas the green bars indicate time horizons at which PRS was cost-effective at a willingness-to-pay threshold of SGD 75,000/QALY. ASCVD = atherosclerotic cardiovascular disease; PCE = pooled cohort equation; PRS = polygenic risk score; QALY = quality-adjusted life year.

One-way deterministic sensitivity analysis indicated that the effects of statin therapy across PRS strata, baseline ASCVD risks, and adherence to statin therapy exerted the greatest influence on the results. Higher baseline ASCVD risk increased the number of ASCVD events and deaths averted, thereby improving cost-effectiveness. In contrast, a higher relative risk under statin therapy, corresponding to a smaller treatment effect, reduced the number of ASCVD events prevented and therefore worsened cost-effectiveness.

### Interpretation in context

These findings have important clinical and policy implications in Singapore, where statin initiation for individuals at intermediate ASCVD risk is currently guided by conventional risk-enhancing factors rather than genetic risk.^46^ However, a subset of these individuals remains at substantially elevated genetic risk and may benefit disproportionately from earlier intervention. Unlike conventional risk calculators, which rely on clinical risk factors that change over time, PRS is genetically determined and remains fixed over the life course.^47^ Incorporating PRS testing enables reclassification of a proportion of this intermediate-risk group into a higher-risk category in which statin therapy is clinically indicated, thereby improving the efficiency of prevention strategies.^8^^,^^9^

In the scenario analysis using the PREVENT equation, the PRS-guided strategy was associated with a lower ICER than in the corresponding PCE-based analysis. This difference was driven primarily by differences in how PREVENT and PCE classify individuals into cardiovascular risk categories. PREVENT generally estimates lower 10-year ASCVD risk than PCE, particularly among older age groups.^48^ As a result, the population classified as intermediate risk differed between the 2 approaches. These differences influence both the number of individuals receiving preventive treatment and the baseline risk of ASCVD events within the target population. In our model, the PREVENT-defined intermediate-risk population had a higher baseline annual probability of ASCVD events than the corresponding PCE-defined population (0.0164 vs 0.010), which translated into a larger absolute benefit from statin therapy. Consequently, more ASCVD events and ASCVD deaths were averted, leading to greater QALY gains and lower incremental costs than in the corresponding PCE-based analysis. This translated into a more favorable cost-effectiveness profile for the PRS strategy under PREVENT.

Although PRS-guided prevention entails higher upfront costs for genotyping, statin therapy, and additional primary-care consultations, these costs are offset over time as avoided MI and stroke events accumulate. PRS can be applied at a younger age, for instance patients in their 20s and 30s, compared with risk calculators like PCE or PREVENT, which are usually applied at age 40 for 10-year risk. As a result, PRS can alert the patient of their ASCVD risk much earlier. This underscores the importance of assessing the clinical utility of genomic screening programs over extended time horizons to capture their full preventive value.

We incorporated statin adherence in the base-case analysis to better reflect real-world treatment effectiveness, and we further examined its influence in deterministic sensitivity analysis. Adherence was one of the parameters with the greatest influence on cost-effectiveness, indicating that long-term persistence with preventive therapy is an important determinant of the value of PRS-guided statin initiation.

In clinical practice, nonadherence to statins is often driven by concerns about side-effects, particularly statin-associated muscle symptoms.^49^ Although observational studies and registries report the incidence of statin-associated muscle symptoms in 5% to 20% of users, randomized controlled trials report an incidence <5%.^50^ Most patients experiencing mild muscle symptoms can continue statin therapy at a reduced dose or switch to an alternative statin; in a small subset where statin intolerance is confirmed, other lipid-lowering strategies may be considered in consultation with a specialist. Severe adverse effects, such as myopathy with markedly elevated creatine kinase or rhabdomyolysis, are extremely rare (approximately 1 in 10,000 patients), as is clinically significant hepatotoxicity (0.001%). Statins may also slightly increase the risk of new-onset diabetes mellitus in susceptible individuals.^2^ Overall, the available evidence suggests that the preventive benefits of targeted statin use outweigh the potential harms in this intermediate-risk population, supporting the feasibility of PRS-guided statin initiation.

Our findings are consistent with international evidence suggesting that the cost-effectiveness of PRS-guided statin therapy emerges only over longer time horizons. For example, a Canadian study using UK Biobank data found that PRS-informed statin initiation in intermediate-risk groups offers good value compared with conventional risk tools.^10^ Similarly, a U.S. payer-perspective study found PRS-guided workplace cardiovascular prevention programs to be feasible and economically favourable.^18^ Across these studies, as in ours, the short-term costs of genotyping initially outweigh the modest reductions in events, but long-term modeling consistently favors targeted treatment based on genetic risk.

Differences in the magnitude of cost-effectiveness estimates between Singapore and Western countries likely reflect Singapore's relatively high hospitalization costs for ASCVD, more conservative statin prescribing practices, and differences in baseline cardiovascular risk.^46^ A unique challenge in Singapore is its multiethnic population. Because most PRS models were derived from European cohorts, their predictive calibration may be less precise among Asian subgroups, with implications for both equity and real-world effectiveness. This underscores the need for local validation datasets before widespread clinical implementation. To address this need for diverse datasets, a 100,000-person Asian population reference cohort using high-throughput sequencing (the SG100K initiative) is currently underway in Singapore.^14^

### Policy implications

Our results provide an economic rationale for incorporating PRS into cardiovascular disease prevention strategies for individuals at intermediate risk in Singapore. At the time of this study, PRS has not been incorporated into standard clinical practice, though it is approaching clinical translation. Importantly, a moratorium is in place preventing insurance companies from using PRS results to raise premiums, providing protection for individuals who undergo testing. Its adoption would align with the National Precision Medicine Programme, which aims to develop population-specific genomic tools for preventive health.^15^ Successful implementation, however, would require several systemic adjustments.

1) Testing infrastructure and integration: Ensure that PRS testing can be offered seamlessly at scale and made accessible to the general population, potentially supported by central laboratories or polyclinic integration.
2) Clinician education: Equip primary care physicians to interpret and communicate PRS results alongside conventional chronic disease risk factors.
3) Patient behavior: Evaluate the impact of PRS disclosure on preventive behavior among target populations.^51^
4) Equity considerations and reimbursement: Provide subsidies or financial support to ensure equitable access to genomic testing among multiethnic populations.
5) Ethical safeguards: Maintain strong data-protection standards, governance structures, and transparent communication with patients about the meaning and limitations of PRS.

Given the long time horizon over which benefits accrue, policymakers should view PRS as a long-term investment in prevention rather than a short-term cost-saving measure.

### Study limitations

First, the transferability of model inputs to Singapore's multiethnic population represents an overarching source of uncertainty. The PCE was used to define the intermediate-risk target population, but we did not perform an independent recalibration or external validation against Singapore-specific outcome data. The hazard ratios for ASCVD risk elevation in high-PRS individuals (HR: 1.62) and for statin efficacy in this group (HR: 0.56) were derived predominantly from European-ancestry populations, and their magnitude may differ in Asian individuals. The long-term predictive validity of current PRS models in Asian populations also remains underexplored. Critically, these 3 uncertainties, in risk classification, risk elevation, and treatment effect, are not fully independent, and all converge on the same model output: the absolute ASCVD benefit of PRS-guided statin initiation. Our sensitivity analyses address each source separately; notably, substituting an Asian-derived risk HR from a large Chinese prospective cohort study (Lu et al;^44^ HR: 1.84) did not materially alter the main conclusions. Nevertheless, the compounded effect of all 3 uncertainties acting simultaneously may exceed what is captured within the probabilistic sensitivity analysis uncertainty intervals. Local validation of both the PCE and CVD-PRS in Singapore's population is currently being addressed through the SG100K initiative and remains an important prerequisite for clinical implementation.

Second, we relied on cost estimates from other countries where Singapore-specific data were unavailable. MI costs were partly derived by applying a South Korean cost ratio to Singapore hospitalization data, which may introduce cross-country transfer bias. We addressed this by applying a wider variance (±50%) to the MI cost parameter in the probabilistic sensitivity analysis, and sensitivity analyses did not materially alter the main conclusions.

Third, our cohort-based state-transition Markov model assumes memoryless transitions. To better reflect recurrent disease progression, we incorporated differential incidence risks for second and third ASCVD events. Nevertheless, a patient-level microsimulation or semi-Markov model would provide greater flexibility in modeling duration dependence and individual heterogeneity; such an approach was beyond the scope of this analysis given the lack of suitable longitudinal data for the target population.

Fourth, although statin adherence was incorporated in the base-case analysis and examined in sensitivity analyses, real-world adherence may be lower and may vary across demographic subgroups. Given the limited published data, we did not model dynamic statin adherence trajectories over time.

Fifth, our model assumed that the top 20% of the PRS distribution would benefit most from early statin initiation. We were unable to explore alternative PRS thresholds to determine how initiating statins for a smaller or larger proportion of patients might affect outcomes.

Sixth, our analysis did not explore subgroup heterogeneity by sex or ethnicity. Because ASCVD risk differs by sex, differences in sex composition between Singapore's population and the source populations for model parameters may affect overall outcomes.

Finally, broader societal costs such as productivity losses and caregiving were excluded, which may underestimate the total economic benefit of avoided ASCVD events.

## Conclusions

This cost-effectiveness analysis suggests that incorporating a cardiovascular polygenic risk score into conventional ASCVD risk stratification in Singapore is unlikely to be cost-effective in the short to medium term but may become cost-effective over longer time horizons as the benefits of avoided ASCVD events accumulate. At 30 years, PRS-guided statin initiation was associated with an ICER of SGD 60,334/QALY, below the SGD 75,000/QALY threshold. These findings suggest that PRS-guided prevention may represent a cost-effective approach to refining statin initiation among intermediate-risk individuals, aligning with Singapore's long-term precision-health strategy. Further research is needed to validate the predictive performance of PRS in local populations and to address implementation, equity, and ethical considerations before broad policy adoption.

## Funding Support and Author Disclosures

The study was supported under the Personalized Cardiometabolic Risk Management Program, sponsored by the Ng Teng Fong Foundation, the Strategic Research Program at Tan Tock Seng Hospital, and the National Healthcare Group. The authors have reported that they have no relationships relevant to the contents of this paper to disclose.
